# Iron depletion suppresses mTORC1-directed signalling in intestinal Caco-2 cells *via* induction of REDD1

**DOI:** 10.1016/j.cellsig.2016.01.014

**Published:** 2016-05

**Authors:** Ailsa Watson, Christopher Lipina, Harry J. McArdle, Peter M. Taylor, Harinder S. Hundal

**Affiliations:** a Division of Cell Signalling & Immunology, School of Life Sciences, University of Dundee, Dundee DD1 5EH, UK; b The Rowett Institute of Nutrition and Health, University of Aberdeen, Bucksburn, Aberdeen AB21 9SB, UK

**Keywords:** DFO, deferoxamine, REDD1, regulated in DNA damage and development 1, mTOR, mammalian target of rapamycin, 4E-BP1, eIF4E-binding protein 1, HIF, hypoxia-inducible factor, PP2A, protein phosphatase 2A, IRS, insulin receptor substrate, PI3K, phosphatidylinositol 3-kinase, S6K1, ribosomal protein S6 kinase 1, S6, ribosomal protein S6, DMT-1, divalent metal transporter-1, TfR, transferrin receptor, S6K1, 4E-BP1, Akt, PP2A, TSC2, Rheb, Deferoxamine

## Abstract

Iron is an indispensable micronutrient that regulates many aspects of cell function, including growth and proliferation. These processes are critically dependent upon signalling *via* the mammalian or mechanistic target of rapamycin complex 1 (mTORC1). Herein, we test whether iron depletion induced by cell incubation with the iron chelator, deferoxamine (DFO), mediates its effects on cell growth through mTORC1-directed signalling and protein synthesis. We have used Caco-2 cells, a well-established *in vitro* model of human intestinal epithelia. Iron depletion increased expression of iron-regulated proteins (TfR, transferrin receptor and DMT1, divalent metal transporter, as predicted, but it also promoted a marked reduction in growth and proliferation of Caco-2 cells. This was strongly associated with suppressed mTORC1 signalling, as judged by reduced phosphorylation of mTOR substrates, S6K1 and 4E-BP1, and diminished protein synthesis. The reduction in mTORC1 signalling was tightly coupled with increased expression and accumulation of REDD1 (regulated in DNA damage and development 1) and reduced phosphorylation of Akt and TSC2. The increase in REDD1 abundance was rapidly reversed upon iron repletion of cells but was also attenuated by inhibitors of gene transcription, protein phosphatase 2A (PP2A) and by REDD1 siRNA — strategies that also antagonised the loss in mTORC1 signalling associated with iron depletion. Our findings implicate REDD1 and PP2A as crucial regulators of mTORC1 activity in iron-depleted cells and indicate that their modulation may help mitigate atrophy of the intestinal mucosa that may occur in response to iron deficiency.

## Highlights

• Cellular iron (Fe) depletion dramatically reduces growth of intestinal Caco-2 cells.
• mTORC1-directed signalling and protein synthesis are reduced in Fe-depleted cells.
• Fe deficiency induces expression and gain of REDD1 in a PP2A-dependent manner.
• PP2A inhibition blocks REDD1 gain and restores mTORC1 activity in Fe-depleted cells.

## 1 Introduction

Iron is an essential micronutrient that plays a vital role in the control of multiple cellular processes including the transfer of electrons within the mitochondrial respiratory chain and the expression of key genes that influence cellular metabolism, growth and differentiation. At the systemic level, iron is essential for the inter-organ transport and tissue uptake of oxygen by virtue of its crucial role in haemoglobin and myoglobin biology. The physiology and control of such processes is thus likely to be affected by changes in iron homeostasis, which, in the absence of a regulated excretory pathway, is critically dependent upon dietary iron intake and mechanisms controlling iron storage and use in tissues such as liver, muscle and erythrocytes [1]. Whilst excess iron accumulation is a feature of genetic disorders such as hereditary haemochromatosis, a far more prevalent problem with respect to iron balance is iron deficiency, which affects nearly 30% of the global population [2].

Growth of mammalian cells is sensitive to iron availability [3], [4], [5] and, as such, this may be an important determinant of the proliferative capacity of cells that normally “turn-over” very rapidly, such as the absorptive epithelial cells lining the intestinal mucosa. A key metabolic pathway which has been implicated strongly as a regulator of cell growth and proliferation is that involving the mammalian or mechanistic target of rapamycin (mTOR) [6]. Reduced mTOR signalling has been reported in cells and tissues of animals rendered iron deficient [17], although the mechanism(s) underlying these observations remains unclear.

mTOR is an essential component of two separate protein signalling complexes, known as mTORC1 and mTORC2, which, owing to their distinct biochemical composition [6], [7], [8], display differential sensitivity to the immunosuppressant rapamycin [6] that only targets mTORC1. Functionally, mTORC2 serves as one of the two upstream kinases involved in the insulin-dependent activation of the serine/threonine kinase PKB (*aka* Akt). In contrast, mTORC1 integrates mitogenic and nutrient signals to ensure that growth and proliferation of cells only occurs under nutritionally favourable conditions — a role made possible by the fact that mTORC1 is activated under amino acid (AA) sufficient conditions (thus promoting phosphorylation of downstream effectors, such as p70S6 kinase 1 (S6K1) and 4E-BP1 that play important roles in the regulation of protein synthesis [9]) but is dramatically repressed upon AA withdrawal [6]. Activation of mTORC1 is crucially dependent upon a small G-protein called Rheb, which in its GTP-loaded “on” form is a potent activator of mTORC1 [10]. The relative amounts of Rheb in the GTP “on” or GDP “off” form depend upon its intrinsic GTPase activity, which is a target for the GTPase-activating protein (GAP) activity of the tuberous sclerosis complex (TSC1/2) [10]. TSC2 is a physiological substrate for PKB/Akt, whose activation by insulin and growth factors induces phosphorylation of TSC2 and inhibition of its GAP activity, which then aids accumulation of active Rheb and a consequential increase in mTORC1 activity [11]. Activation of mTORC1 is also dependent on small G proteins of the Rag family, which operate as heterodimers (RagA or RagB with RagC or RagD) to promote redistribution of mTORC1 to lysosomal membranes in response to AA provision [12]. Rags are tethered to the lysosomal surface by interactions with two heteromeric protein complexes; (i) the Ragulator (Rag regulator) complex [12] and (ii) the vacuolar H^+^-ATPase resident in the lysosomal membrane [13]. AA-dependent modulation of these interactions appears to facilitate binding of mTORC1 to Rag complexes, placing it in close proximity to its activator Rheb [13].

In contrast, inactivation of mTOR may, in part, be driven by regulating the localisation of the TSC complex. Insulin and AAs have recently been shown to promote dissociation of TSC1/TSC2 from lysosomal membranes, whereas the absence of these stimuli induces greater lysosomal association of the complex where it facilitates conversion of Rheb to its inactive GDP-form and thus a reduction in mTOR activity [14], [15]. mTORC1 can also be negatively regulated by REDD1 (regulated in DNA damage and development 1), a small 25 kDa protein whose expression is induced in response to environmental stresses, such as hypoxia [16]. Precisely how REDD1 inhibits mTORC1 activity is unclear although it has been suggested to sequester 14-3-3 proteins away from TSC2, which may then permit TSC2 to target its GAP activity towards Rheb [17]. More recent work has shown that ectopic over-expression of REDD1 in HEK293 cells induces association of protein phosphatase 2A (PP2A) with Akt causing dephosphorylation and inactivation of the kinase on one of its key regulatory sites (Thr308) that, in turn, reduces its capacity to phosphorylate and inhibit TSC2 and consequently promote downstream activation of Rheb [18]. However, it remains unclear if such a mechanism may account for the reduction in Akt and mTORC1 signalling observed in cells and tissues of animals rendered iron deficient [17]. In this study we have investigated the effect of iron deficiency on the growth and proliferative potential of intestinal epithelial cells. We show that iron depletion induced in human intestinal Caco-2 cells by treatment with the iron chelator deferoxamine (DFO) results in REDD1 induction and that this is associated with not only a fall in Akt and TSC2 phosphorylation, but reduced mTORC1 signalling and a marked suppression in protein synthesis and cellular proliferation. Strikingly, the increase in REDD1 expression initiated by DFO treatment can be attenuated by PP2A inhibition and this is associated with retention of mTORC1 signalling in otherwise iron-deficient cells. Our work identifies REDD1 and PP2A as potential therapeutic targets whose modulation may help restrain loss in mTORC1 activity and thereby potentially limit intestinal mucosal atrophy associated with chronic or recurrent iron deficiency disorders [19], [20].

## 2 Materials and methods

### 2.1 Materials

Dulbecco's Modified Eagle Medium (DMEM), penicillin–streptomycin, MEM Non-Essential AAs Solution, amphotericin B and Trypsin–EDTA were purchased from Life Technologies (Paisley, UK). Heat inactivated foetal bovine serum (FBS) was bought from Thermo Scientific (Waltham, MA, USA). Sodium pyruvate, Earle's Balanced Salt Solution (EBSS), gelatin from cold water fish skin (FSG), TriReagent®, SYBR® Green kit, human holo-Transferrin and all protease inhibitors were purchased from Sigma-Aldrich (Poole, UK). Puromycin and cycloheximide was purchased from Abcam (Cambridge, UK). Protein Assay Dye Reagent Concentrate was from Bio-Rad (Hemel Hempstead, UK). GoTaq® DNA Polymerase, deoxyribonucleotide phosphates (dNTPs) and Moloney Murine Leukaemia Virus (M-MLV) reverse transcriptase were from Promega (Southampton, UK). Deferoxamine (DFO) mesylate salt and polyvinylidene difluoride (PVDF) membranes were purchased from Merck Millipore (Darmstadt, Germany). X-ray film was sourced from Konica Minolta (Tokyo, Japan). The transcription inhibitor actinomycin D was purchased from Tocris (Bristol, UK) and the proteasome inhibitor MG132 was from Selleck Chemicals (Houston, USA). Antibodies to DMT1, TfR and PP2A-α (Tyr 307) were purchased from Abcam (Cambridge, UK). Actin and GAPDH antibodies were from Sigma (Poole, UK). The antibody to HIF-1α was purchased from BD Biosciences (Oxford, UK), the REDD1 antibody from Proteintech (Manchester, UK), the puromycin antibody from Kerafast (Boston, USA) and native PP2A from the Division of Signal Transduction Therapy (DSTT; University of Dundee, UK). All other antibodies, including horse-radish peroxidise-conjugated secondary antibodies (mouse and rabbit) and that to cleaved caspase 3, were purchased from New England Biolabs (Hitchin, UK). All qPCR primers were synthesised by the Oligonucleotide Synthesis Service (University of Dundee).

### 2.2 Cell culture

Caco-2 cells were cultured in Dulbecco's Modified Eagle Medium (DMEM) containing 1 mM sodium pyruvate, 1% (v/v) penicillin–streptomycin (final concentration 100 μg/ml penicillin, 100 μg/ml streptomycin), 1% (v/v) non-essential AAs, 10% (v/v) heat inactivated foetal bovine serum (FBS) and 250 ng/ml amphotericin B. Medium was changed every two days and cells were maintained at 37 °C in 10% CO_2_. For experimentation, cells were seeded at a density of 2.2 × 10^4^ cells/cm^2^ on 6-well plates and reached confluence by day 3. Caco-2 cells were depleted of iron by incubation in media supplemented with 10% FBS and the iron chelator deferoxamine (DFO) [5] for periods and at concentrations indicated in the text and figure legends.

### 2.3 Protein extraction, immunoprecipitation and immunoblotting

Immunoblot analysis was carried out as previously described [21]. Following cell treatment, Caco-2 cells were washed with ice cold PBS and then immediately lysed in ice cold lysis buffer [50 mM Tris/HCl pH 7.4, 0.27 M sucrose, 1 mM sodium orthovanadate, 1 mM EDTA, 1 mM EGTA, 10 mM sodium 2-glycerophosphate, 50 mM sodium fluoride, 5 mM sodium pyrophosphate, 1% (w/v) Triton X-100, 0.1% (v/v) 2-mercaptoethanol and protease inhibitor (one tablet/50 ml)]. Cell debris was removed from crude cell lysates by centrifugation at 3000 *g* for 10 min at 4 °C, and the resulting supernatant used for Western blot analysis. In some experiments, following treatment with DFO, Caco-2 cells were lysed as described above. PP2Ac was immunoprecipitated from 0.5 mg of protein lysate using an antibody against the C-terminal domain of PP2Ac. Resulting immunocomplexes were captured by incubation with protein-A-sepharose beads and solubilised in Laemmli sample buffer prior to immunoblotting using an antibody targeting REDD1 or PP2Ac. Proteins from cell lysates (30 μg) or immunoprecipitates were subjected to SDS-polyacrylamide gel electrophoresis and immunoblotted using primary antibodies to proteins of interest as previously described [21]. Resulting band intensities were quantified using ImageJ software (National Institutes of Health, Bethesda, MD).

### 2.4 RNA extraction and qPCR analysis

Total RNA was extracted from Caco-2 cells using Tri-Reagent® according to manufacturer's instructions (Sigma-Aldrich). Quantitative real-time PCR was performed using a StepOne Plus Real-Time PCR System (Applied Biosystems), SYBR Green JumpStart Taq Ready Mix (Sigma-Aldrich) and primers targeting 18S rRNA (18S ribosomal ribonucleic acid) as a control. The human sequences for the primers used were as follows: DMT1 forward, 5′-GACTCGCTCTATTGCCATCATCC-3′; DMT1 reverse, 5′-ATCCGCCAGCCTAGTCCATTG-3′; TfR forward, 5′-CAATACAGAGCAGACATAAAGGAA-3′; TfR reverse, 5′-CTGGAAGTAGCACGGAAGAA-3′; REDD1 forward, 5′-CTTTGGGACCGCTTCTCGTC-3′; REDD1 reverse, 5′-GGTAAGCCGTGTCTTCCTCCG-3′; 18S rRNA forward, 5′-CAGCCACCCGAGATTGAGCA-3′; 18S rRNA reverse, 5′-TAGTAGCGACGGGCGGTGTG -3′. qPCR amplification was performed with an initial denaturation at 95 °C for 10 min followed by 40 cycles of denaturation at 95 °C for 15 s, annealing at 55 °C for 15 s, and extension at 68 °C for 1 min. The ratio of mRNA of interest to 18S rRNA mRNA expression was calculated using a mathematical model previously described [22].

### 2.5 Amino acid analysis by HPLC

Caco-2 cells were lysed in water and mixed with trifluoroacetic acid and methanol (1 ∶ 10). Supernatants were dried off in a rotary evaporator at 46 °C. Samples were suspended in sodium acetate, Methanol, TEA (2 ∶ 2:1), methanol, H_2_O, TEA, PITC (7 ∶ 1:1 ∶ 1) and methanol (100%) with drying steps between each step. The resulting phenylthiocarbamyl peptides were separated by a Hewlett Packard 1050 HPLC system (Minnesota, USA) with post-column UV detection (254 nm). HPLC traces were analysed using the Clarity Lite software.

### 2.6 Protein synthesis

Protein synthesis was measured as described by Goodman et al. [23] by assaying the incorporation of puromycin into newly synthesised peptides. Briefly, cells were pretreated as described in the figure legend with DFO, iron-loaded DFO or cycloheximide (50 μg/ml) prior to incubation in the absence or presence of 1 μM puromycin for 30 min. At the end of this period cells were lysed and lysates subjected to SDS-PAGE and immunoblotting of PVDF membranes carried out overnight at 4 °C with a mouse monoclonal anti-puromycin antibody (1 μg/ml in TBST with 5% w/v non-fat dry milk) followed by incubation with goat anti-mouse HRP secondary antibody.

### 2.7 Statistical analysis

Statistical analysis was performed using GraphPad Prism. Data are presented as mean ± standard error of mean (SEM) with quantitative and statistical assessments being derived from a minimum of three replicates per group. Values used to normalise results in each experiment were excluded from statistical analysis, which involved analysis of variance (ANOVA) and a Newman–Keuls post-test for multiple comparisons. Significance was set at a *P* value of < 0.05.

## 3 Results

### 3.1 Effects of iron deficiency on proliferation of Caco-2 cells

**Fig. 1:**
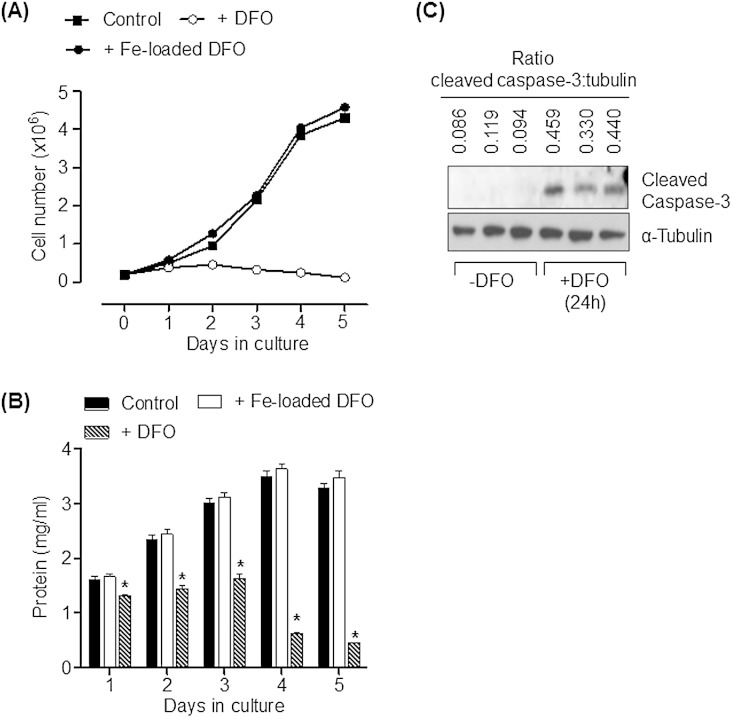
Effects of DFO-induced iron depletion upon cellular proliferation in Caco-2 cells. (A) Caco-2 cells were seeded at a density of 200,000 cells/cm^2^. Following adherence to the culture plate cells were treated with or without 100 μM DFO or 100 μM iron-loaded DFO for up to 5 days with media and treatment agent being refreshed every 24 h. Cell number was assessed every 24 h during the 5 day treatment period. Point values on the line graphs for each treatment represent mean ± SEM from three experiments. (B) Cells were treated in (A) and each analytical time point was lysed for analysis of cell protein. The asterisk represents a significant change (p < 0.05) from the control bar value on each of the treatment days. Bars represent mean uptake ± SEM from three experiments. (C) Caco-2 cells were incubated with 100 μM DFO 24 h (n = 3) or vehicle solution (n = 3) prior to cell lysis and immunoblot analysis using antibodies targeting cleaved caspase 3 and α-tubulin (as a gel loading control). The ratio of cleaved caspase 3 to α-tubulin is indicated above each lane.

Monolayers of Caco-2 cells were iron depleted by exposure to 100 μM DFO (an iron-chelator). Fig. 1A shows that, compared to control cells or cells incubated with iron-loaded DFO, sustained exposure of Caco-2 cells to DFO effectively suppressed cell proliferation over the treatment period with the chelator. In line with this observation, whilst total cell protein increased in control cells or those incubated with iron-loaded DFO this was not observed in cells that were iron-depleted with DFO (Fig. 1B). Indeed, exposure of cells to DFO for periods greater than 3 days was associated with a reduction in total cell protein which most likely arises as a consequence of iron depletion initiating an increase in cell death. The presence of cleaved caspase 3 (a well-established apoptotic marker) in cells that had been treated with DFO for 24 h is entirely consistent with this latter proposition (Fig. 1C).

### 3.2 Iron deficiency downregulates mTORC1-directed signalling

**Fig. 2:**
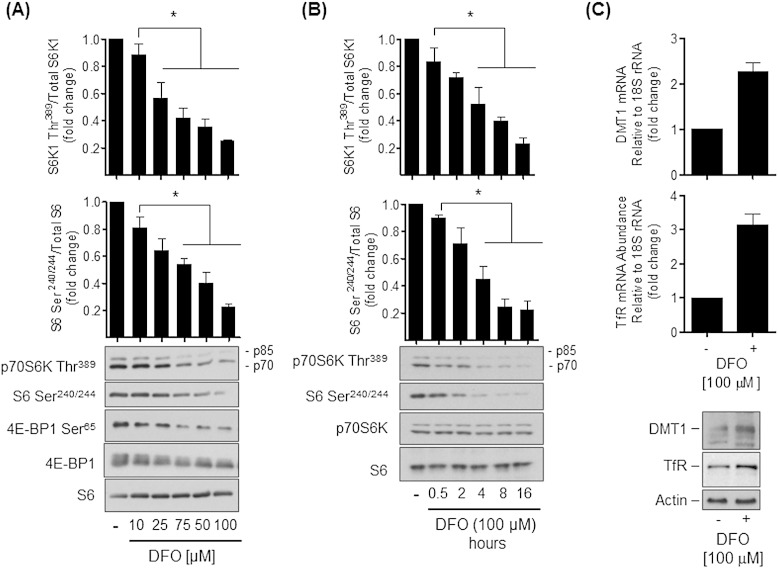
Iron depletion suppresses mTOR signalling and enhances mRNA and protein expression of TfR and DMT-1. Caco-2 cells were incubated with deferoxamine (DFO) at concentrations indicated in (A) for 16 h or with 100 μM DFO for incubation periods as indicated in (B) prior to cell lysis and immunoblot analysis using antibodies targeting p70S6 Kinase Thr^389^, S6 Ser^240/244^, 4E-BP1 Ser^65^, 4E-BP1 and S6. Immunoblot data was quantified using ImageJ and presented as mean ± SEM from five independent experiments. Asterisks denote statistical significance between the indicated values (*p < 0.05). (C) Total RNA was extracted from Caco-2 cells that had been treated with or without 100 μM DFO for 16 h and DMT1 and TfR mRNA expression assessed using real-time PCR by normalising to 18S rRNA. Lower panel shows the effect of DFO treatment in these cells upon DMT1 and TfR protein abundance.

The mTORC1/S6K1 signalling axis exerts a positive influence on cell growth and protein synthesis. Consequently, impaired activation of this signalling pathway in iron depleted cells would be expected to impact negatively on these cellular processes. In an attempt to understand the impact that iron deficiency may have upon mTORC1 signalling, Caco-2 cells maintained in DMEM containing amino acids and FBS were incubated with increasing concentrations of DFO (0–100 μM) for 16 h prior to assessing the phosphorylation status of S6K1, S6 and 4E-BP1. Fig. 2A shows that in the absence of DFO treatment we observed phosphorylation of S6K1 and 4E-BP1 on their respective mTORC1 regulated sites (Thr^389^ and Ser^65^) and that of ribosomal S6 on Ser^240/244^, which represent downstream target sites for S6K1. Phosphorylation of all three proteins was reduced by cell treatment with DFO in a dose-dependent manner with that of S6K1 and S6 being suppressed by up to ~ 80% in response to a 100 μM DFO dose. At this dose, phosphorylation of S6K1 and S6 were maximally reduced by 16 h of cell treatment with DFO (Fig. 2B). It is noteworthy that multi-site 4E-BP1 phosphorylation normally results in the appearance of a diffuse immunoreactive protein band for total 4E-BP1 as shown in the control lane, which stems from the differential gel shifts associated with varying 4E-BP1 phosphorylation. However, consistent with its dephosphorylation in iron-depleted cells this 4E-BP1 band becomes sharper with increasing concentrations of DFO. It is also important to stress that the reduced phosphorylation of S6K1, S6 or 4E-BP1 induced by DFO treatment is not a consequence of changes in the overall expression of these proteins, which were not significantly affected by DFO.

The reduction in mTORC1 signalling seen with the DFO in our cells is within the dose range and incubation times previously reported by other investigators exploring effects of iron depletion on, for example, cellular signalling and growth arrest [5], [24]. However, to validate that DFO does indeed reduce iron availability within our cells we monitored the abundance of the divalent metal transporter-1 (DMT-1) and the transferrin receptor (TfR), whose expression is known to be upregulated in cells and tissues upon iron depletion [25], [26], [27]. Fig. 2C shows that Caco-2 cells incubated with DFO (100 μM, 16 h) exhibit a 2.3 and 3-fold increase in DMT-1 and TfR mRNA expression respectively, which is also associated with increased cellular abundance of both proteins (Fig. 2C, lower panel).

### 3.3 Effects of DFO treatment on AA-induced activation of S6K1 and intracellular AA content

**Fig. 3:**
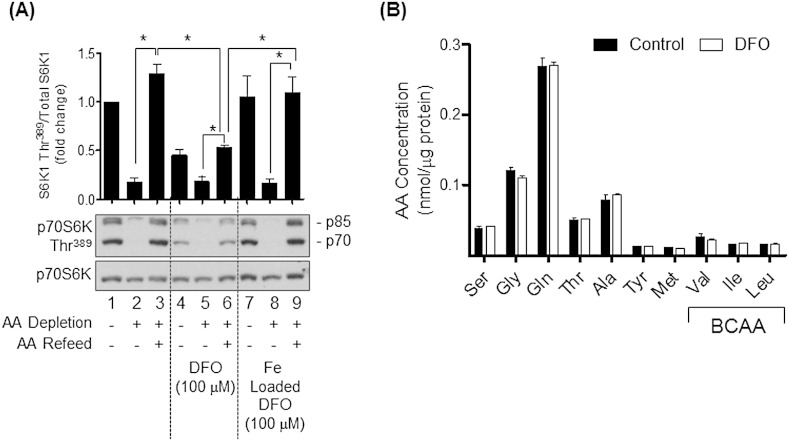
Effects of iron depletion upon amino acid-dependent activation of mTORC1 signalling and intracellular amino acid abundance in Caco-2 cells. (A) Caco-2 cells were incubated in DMEM/10% FBS, in the absence or presence of 100 μM deferoxamine (DFO) or 100 μM iron loaded DFO for 16 h prior to being incubated in Hepes buffered saline (HBS)/10% FBS containing or lacking amino acids (AA) for a further 6 h. In some experiments, cells that had been incubated in AA-depleted buffer were subsequently “refed” with HBS/FBS containing 1x physiological AA mix for 15 min. At the end of these incubations, cells were lysed and lysates used for immunoblotting with antibodies against p70S6 Kinase Thr^389^ and native p70S6 Kinase. Immunoblots were quantified and data presented as mean ± SEM from three experiments. Asterisks indicate a significant change (p < 0.05) between indicated values. (B) Cells were incubated in the absence or presence of 100 μM DFO for 16 h before lysis in water and analyses of AA content by HPLC. Values presented are the mean ± SEM from three independent experiments.

In addition to insulin and growth factors, mTORC1 activation is crucially dependent upon AA availability [6]. Consequently, it is plausible that the loss in S6K1, 4E-BP1 and S6 phosphorylation induced by DFO treatment may be driven by changes in cellular AA balance and/or their capacity for inducing stimulation of the mTORC1/S6K1 signalling axis. To test this proposition, Caco-2 cells that had undergone prior incubation with media containing serum and DFO or the vehicle solution alone were AA deprived for 6 h or having been AA deprived were subsequently held in media containing serum and AAs for 15 min. Analysis of S6K1 phosphorylation revealed that, in the absence of DFO treatment, depriving cells of AA for 6 h reduced S6K1 phosphorylation by over 80% (Fig. 3A, compare lane 1 and 2), whereas acute AA refeeding after a period of AA starvation reinstated S6K1 phosphorylation to a level comparable if not greater than that seen in control cells (compare lane 3 with lanes 1 and 2). Consistent with the data presented in Fig. 2A, DFO treatment reduced S6K1 phosphorylation by ~ 60%, which was reduced further (by ~ 20%) when iron-depleted cells were also AA deprived (Fig. 3A, lanes 4 and 5). Intriguingly, refeeding DFO-treated cells with AA could only partially recover the deficit in S6K1 phosphorylation (compare lanes 3 and 6) suggesting that whilst AA availability was an important determinant of S6K1 activation, iron depletion is associated with anabolic resistance in terms of the ability of AAs to fully activate mTORC1 signalling. It is noteworthy that HPLC analysis of intracellular AA content especially of those AAs (*e.g.* BCAA) that convey a potent stimulatory effect on mTORC1 signalling [6] were found to be unaffected by DFO treatment (Fig. 3B) thus excluding the possibility that reduced mTORC1 signalling by AAs was a consequence of disturbances in the intracellular AA pool.

As an additional control, we also monitored the effects of AA withdrawal/refeeding on S6K1 phosphorylation in cells incubated with DFO that had been preloaded with iron so that the chelator would not induce iron depletion when applied onto cells. Fig. 3A shows that this treatment had no impact upon S6K1 phosphorylation (compare lanes 1 and 7) and that the response to AA withdrawal/refeeding was similar to that seen in non-DFO treated cells (compare lanes 2 and 3 with lanes 8 and 9); *i.e.* the effects were due to iron depletion rather than any off-target effect of DFO.

### 3.4 Cellular iron depletion impairs Akt-mediated signalling

**Fig. 4:**
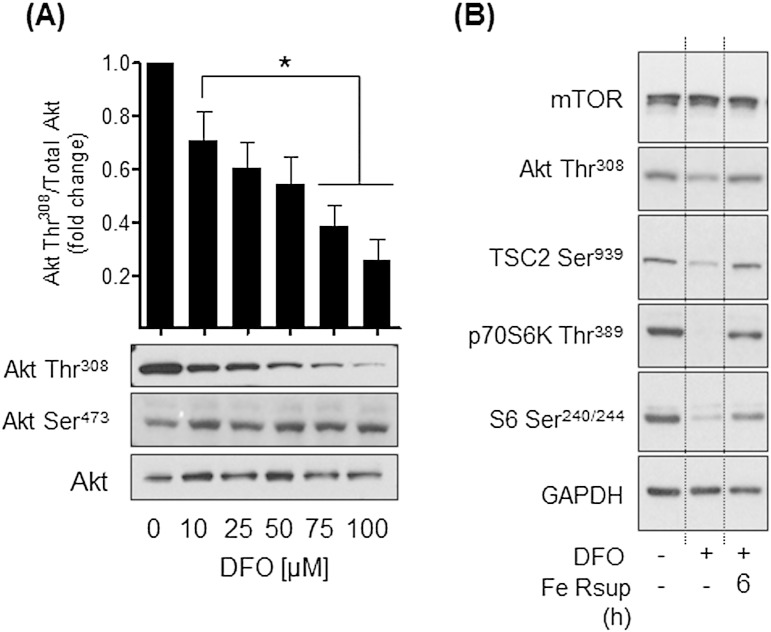
Effects of DFO-induced iron depletion on Akt and mTORC1 signalling in Caco-2 cells. Caco-2 cells were incubated with deferoxamine (DFO) at concentrations indicated in (A) for 16 h. (B) Alternatively, having been incubated with DFO for this period they were subsequently resupplemented with iron using human 10 μM holo-Transferrin for 6 h. At the end of these incubations cells were lysed and lysates immunoblotted with antibodies against (A) Akt Thr^308^, Akt Ser^473^ and native Akt or (B) proteins indicated. The Akt Thr^308^ signal in (A) was quantified and presented as the mean ± SEM from six independent experiments. Asterisks denote statistical significance between the indicated values (*p < 0.05).

In an attempt to understand how iron depletion reduces mTORC1 signalling we subsequently explored the effect of DFO treatment on Akt signalling, which conveys a positive stimulatory input into mTORC1 signalling. Fig. 4A shows that DFO induces a dose-dependent reduction in AktThr^308^ phosphorylation, which fell significantly over a 16 h period by up to 80% in cells treated with 100 μM DFO. Strikingly, we did not observe any detectable changes in AktSer^473^ phosphorylation over the DFO dose range used in these studies. Since full activation of the kinase is dependent on phosphorylation of both Akt regulatory sites [28], we postulated that loss of Thr^308^ phosphorylation would reduce Akt signalling capacity. In line with this idea, Fig. 4B shows that phosphorylation of TSC2 on Ser^939^ (a physiological downstream Akt target site) was notably reduced in DFO-treated cells along with reduced S6K1 and S6 phosphorylation. Resupplementation of iron-depleted cells with iron saturated holo-transferrin for 6 h not only reinstated AktThr^308^ and TSC2Ser^939^ phosphorylation, but also led to an attendant increase in S6K1 and S6 phosphorylation. These latter observations showing reversibility of the effects of iron depletion could not be attributed to changes in mTOR abundance, which was unaffected by modification of cellular iron status (Fig. 4B).

### 3.5 Modulation of REDD1 expression in Caco-2 cells in response to DFO treatment

**Fig. 5:**
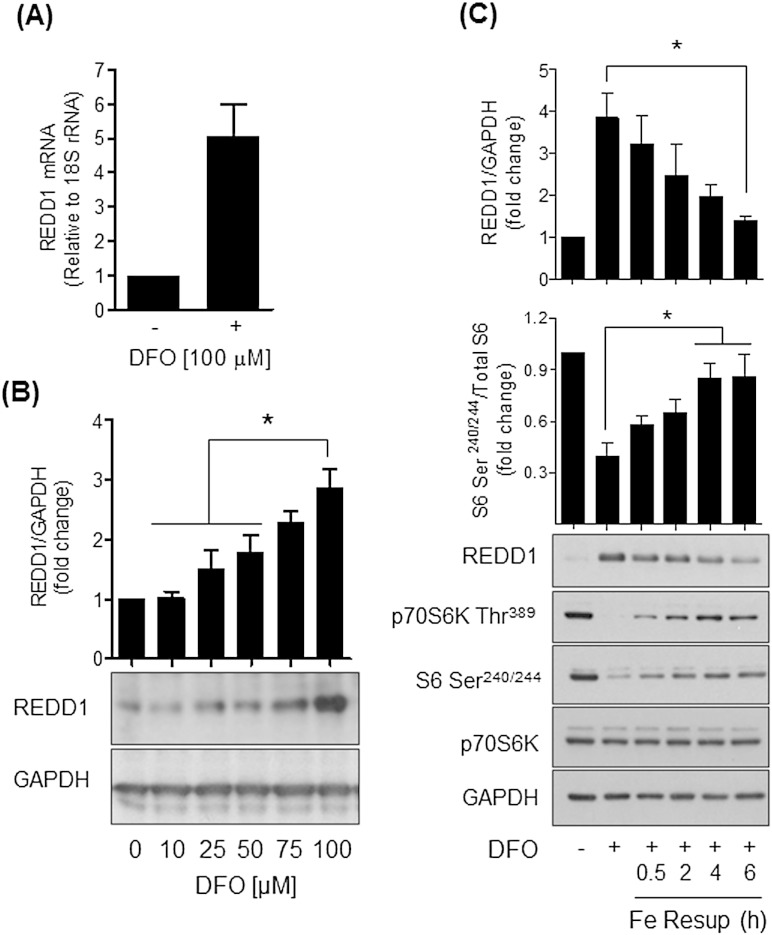
Effects of iron depletion/resupplementation upon REDD1 mRNA and protein expression in Caco-2 cells. Caco-2 cells were incubated in the absence or presence of (A) 100 μM DFO for 16 h or (B) at the concentrations indicated. In some experiments (C), cells treated with DFO were subjected to iron resupplementation using 10 μM holo-Transferrin over a 6 h period. At the end of these treatment incubations total mRNA was extracted from Caco-2 cells and (A) REDD1 mRNA abundance quantified relative to 18S rRNA by qPCR. Alternatively, cells were lysed and lysates immunblotted using antibodies against REDD1, p70S6 Kinase Thr^389^, S6 Ser^240/244^ and GAPDH, which served as a gel loading control. Corresponding quantification for REDD1 and S6 Ser^240/244^ phosphorylation is shown above blots and data expressed as mean ± SEM from at least three experiments. The asterisk represents a significant change (p < 0.05) between indicated values.

REDD1, a negative regulator of mTORC1 signalling, is induced by various environmental stresses and its ectopic over-expression in HEK293 cells was recently shown to also induce a reduction in AktThr^308^ phosphorylation [16]. We therefore explored whether REDD1 may participate in the response to iron depletion observed in our cells. Fig. 5A shows that REDD1 gene expression was elevated by over 5-fold in cells treated with 100 μM DFO and that, over this same dose and incubation period, the iron chelator increased REDD1 protein by ~ 3-fold (Fig. 5B). This DFO-induced increase in REDD1 correlated with a loss in S6K1 and S6 phosphorylation and was repressed upon resupplementing DFO-treated cells with iron in a time-dependent manner (Fig. 5C).

### 3.6 A role for PP2A in the regulation of REDD1 expression and mTORC1 signalling in iron-depleted cells

**Fig. 6:**
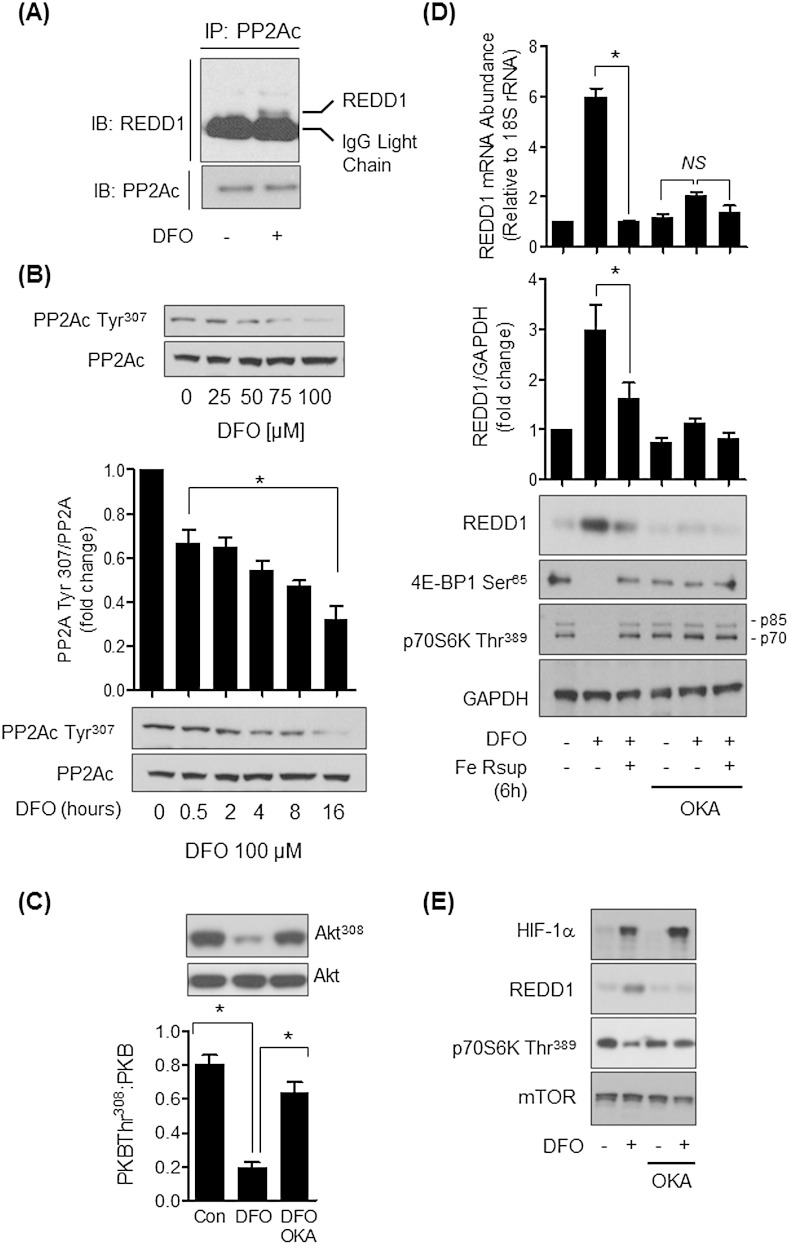
Effects of DFO and okadaic acid on PP2A tyrosine phosphorylation, REDD1 expression and mTORC1 signalling in Caco-2 cells. (A) Caco-2 cells were incubated with DMEM/10% FBS media supplemented with DFO (100 μM) for 16 h prior to immunoprecipitation of PP2Ac and immunoblotting with antibodies to REDD1 or PP2Ac. (B) Cells were incubated as in (A) but at the indicated DFO concentrations for 16 h or with 100 μM DFO for times indicated after which cells were lysed and immunoblotted with antibodies against phospho-Tyr^307^ of PP2Ac or native PP2Ac. (C) Cells were incubated with vehicle or 100 μM DFO for 16 h or with okadaic acid (OKA, 100 nM) for the penultimate 2 h period of treatment with DFO after which cells were lysed and immunoblotted with antibodies against AktThr^308^ or native Akt. (D) Caco-2 cells were incubated in the absence or presence of 100 μM DFO for 16 h, or having been treated with DFO for 16 h resupplemented with human holo-Transferrin for a further 6 h. In some experiments, cells treated with DFO and holo-Transferrin were also incubated with okadaic acid (OKA, 100 nM) for the penultimate 2 h period of treatment with DFO or 10 μM holo-Transferrin. At the end of these incubations cells were either prepared for analysis of REDD1 mRNA content by qPCR analysis (upper panel) or lysed and lysates immunoblotted with antibodies against REDD1 and S6 Ser^240/244^. Analysis of REDD1 mRNA and protein abundance is presented as mean ± SEM from at least three experiments. Immunoblots (B, C and D) were quantified using ImageJ and data presented are the mean ± SEM from three or four independent experiments. The asterisk denotes statistical significance between the indicated values (*p < 0.05). (E) Cells were treated as in (D) with DFO and okadaic acid at the end of which they were and lysates immunoblotted with antibodies to the proteins shown.

Previous work has suggested that REDD1 can interact with PP2A and influence mTORC1 signalling by inducing the dephosphorylation and upstream inactivation of Akt on Thr^308^ [16]. Consistent with these previous observations immunoprecipitation of PP2Ac from Caco-2 cells revealed that we could detect coimmunoprecipitation of endogenous REDD1 in DFO but not in control cells treated with vehicle solution alone. The immunoreactive REDD1 band migrated marginally ahead of the much more intense IgG light chain signal (Fig. 6A). Evidence in the literature indicates that PP2A functions as a negative regulator of Akt and that decreased Tyr^307^ phosphorylation of the catalytic PP2A subunit (PP2Ac) is associated with increased phosphatase activity towards substrates such as Akt [29], [30], [31]. In line with this idea, Fig. 6B shows that PP2Ac Tyr^307^ phosphorylation was reduced in DFO-treated cells in a dose and time-dependent manner and that AktThr^308^ phosphorylation was accordingly reduced in DFO treated cells (Fig. 6C). The notion that dephosphorylation of this Akt site is likely mediated by PP2A is supported by our finding that it was suppressed by okadaic acid, a potent PP2A inhibitor (IC_50_ = 0.1–1 nM [32]), in DFO-treated cells (Fig. 6C). To determine the likely impact of increased PP2A activity upon REDD1 and mTORC1 signalling in DFO-treated cells we further explored the effect of okadaic acid on this signalling axis. Fig. 6D shows that the DFO-induced increase in REDD1 was dramatically attenuated when DFO-treated cells were coincubated with okadaic acid. Under these circumstances, the loss in mTORC1 signalling associated with iron depletion was also ameliorated as judged by analysis of S6K1 and S6 phosphorylation (Fig. 6D and E). Expression of the REDD1 gene can be upregulated by a number of transcription factors, including HIF-1 [33] whose abundance is known to be enhanced in iron-depleted tissues [34]. In line with previous work we find that iron depletion increases HIF1α content in Caco-2 cells. However, whilst induction of REDD1 was sensitive to okadaic acid the increase in HIF1α was not affected by the inhibitor (Fig. 6E).

### 3.7 Effects of modulating REDD1 protein on mTORC1 signalling in DFO-treated cells

**Fig. 7:**
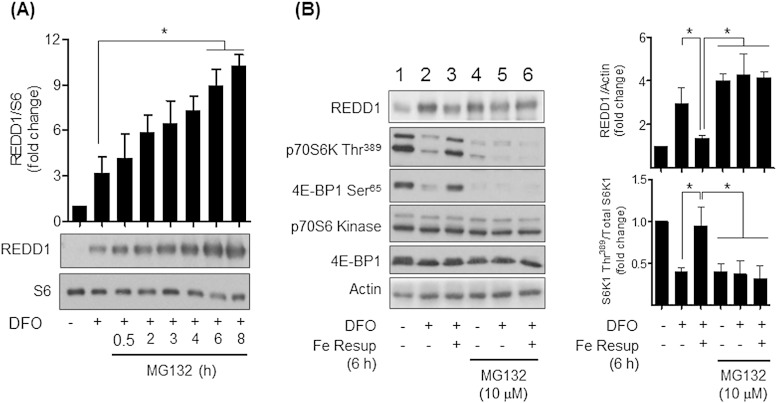
Effects of DFO and proteosomal inhibition on REDD1 expression and mTORC1 signalling in Caco-2 cells. (A) Cells were treated in the absence or presence of 100 μM DFO for 16 h prior to incubation with 10 μM MG132, a proteasomal inhibitor for time periods indicated. At the end of these incubations cells were lysed and immunoblotted with antibodies against REDD1 or native S6. Blots from a minimum of three experiments were quantified and the data presented as mean ± SEM. The asterisk denotes significant differences between the indicated values (*p < 0.05). (B) Caco-2 cells were pre-incubated in the absence or presence of 100 μM DFO for having been treated with DFO for this period subsequently incubated with human holo-Transferrin to resupplement iron to cells. In some experiments, cells were also coincubated with 10 μM MG132 during the duration of the DFO or DFO with + holo-Transferin treatment period. Cells were lysed and immunoblotted for analysis of REDD1 and phosphorylation of p70S6 Kinase Thr^389^, 4E-BP1 Ser^65^, total p70S6 Kinase, 4EBP-1 and actin. REDD1 and p70S6 Kinase Thr^389^ immunoblots were quantified (right bar graph panel) and data presented as mean ± SEM from at least three experiments. The asterisks signify significant differences (p < 0.05) between indicated bar values.

To assess the importance of REDD1 as a modulator of mTORC1 signalling in iron-depleted cells we subsequently investigated the impact of either stabilising REDD1 or substantially reducing its transcriptional expression. REDD1 is a target for the Cul4a-DDB1-ROC1-β-TRCP ubiquitin E3 ligase complex [35] and can be degraded by the ubiquitin-proteosome system [35]. In line with this, in addition to its induction by DFO treatment, the cellular abundance of REDD1 was further enhanced in a time-dependent manner by cell treatment with MG132, a proteosomal inhibitor (Fig. 7A). Proteosomal inhibition in the absence of DFO treatment was also effective in stabilising REDD1 (Fig. 7B, compare lane 1 with 4) suggesting that under normal circumstances the low abundance of REDD1 most likely reflects its rapid turnover within cells. Accumulating REDD1 within cells through proteosomal inhibition also serves to inhibit phosphorylation of S6K1 and 4E-BP1 (Fig. 7B, compare Lanes 1 and 4). Intriguingly, whilst iron resupplementation in DFO-treated cells antagonises REDD1 expression and the loss in S6K1 and 4E-BP1 phosphorylation (Fig. 7B, compare Lanes 2 and 3), this intervention fails to repress accumulation of REDD1 or subvert the loss in mTORC1 signalling in cells incubated with MG132 (Fig. 7B, compare Lanes 5 and 6).

**Fig. 8:**
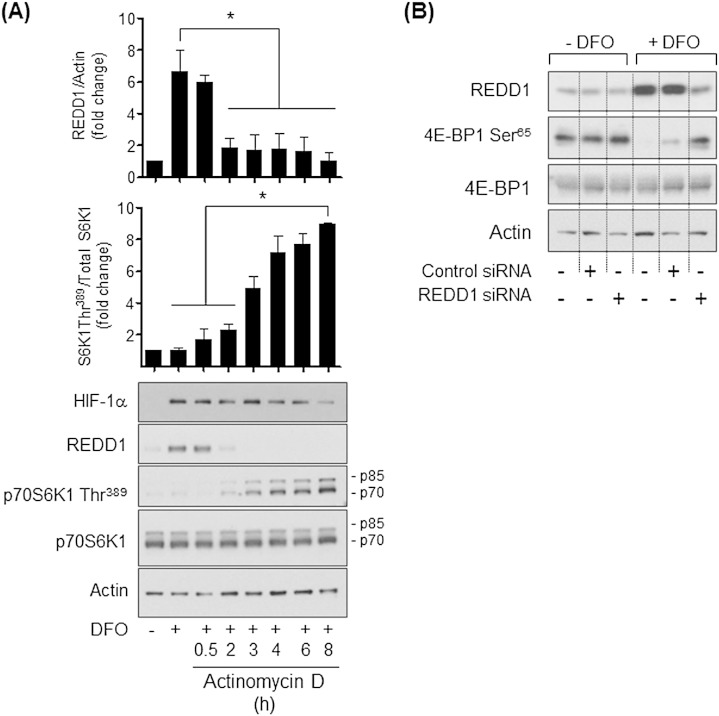
Effects of modulating REDD1 expression on DFO-induced changes in mTORC1 signalling in Caco-2 cells. (A) Caco-2 cells were incubated in the absence or presence of 100 μM DFO for 16 h prior to incubation with 100 μM Actinomycin D, a transcriptional inhibitor for time periods indicated. At the end of these treatments cells were lysed and lysates immunblotted with antibodies against HIF-1α, REDD1, p70S6K Thr^389^, p70S6K and actin. REDD1 and p70S6K Thr^389^ abundance was quantified using ImageJ and data presented as mean ± SEM from at least three experiments. The asterisks signify significant differences (p < 0.05) between indicated bar values. (B) Untransfected Caco-2 cells or those transiently transfected with 1 μg control siRNA or 1 μg REDD1 siRNA for 24 h were incubated in the absence or presence of 100 μM DFO for 16 h. At the end of this treatment cells were lysed and immunoblotted with antibodies against REDD1, 4E-BP1 Ser^65^, 4E-BP1 and Actin. The blots are representative of two separate experiments.

In contrast to stabilising REDD1 protein, treatment of cells with actinomycin D, a transcriptional inhibitor, induced a striking time-dependent reduction in REDD1 protein in DFO-treated cells (Fig. 8A), consistent with the known short half-life of this protein in cells [36], [37]. This decline in REDD1 expression correlated with a corresponding increase in S6K1 phosphorylation despite cells being iron depleted. This latter response could not be attributed to changes in total S6K1 abundance which we found to be unaffected over the actinomycin D incubation period (Fig. 8A). Whilst REDD1 protein content was rapidly reduced by actinomycin D in iron depleted cells it is noteworthy that the increased abundance of HIF1α was sustained for much longer periods in the presence of the transcription inhibitor.

The changes in REDD1 abundance associated with MG132 or actinomycin D treatment shown in Fig. 7, Fig. 8A inversely correlate with what happens to mTORC1 signalling. For more direct evidence that REDD1 is a key determinant of mTORC1 signalling in iron-depleted cells we also monitored the effect of DFO treatment in Caco-2 cells in which REDD1 had been transiently silenced. Fig. 8B shows that 16 h of DFO treatment induces a robust increase in REDD1 protein in untransfected Caco-2 cells or those that had been transiently transfected with a control siRNA, but that this induction was notably muted in cells transfected with siRNA targeting REDD1. Whilst phosphorylation of 4E-BP1 was markedly reduced in the two iron-depleted control groups we were able to detect elevated 4E-BP1 phosphorylation in DFO-treated REDD1 silenced cells.

### 3.8 Effects of DFO treatment on cellular protein synthesis

**Fig. 9:**
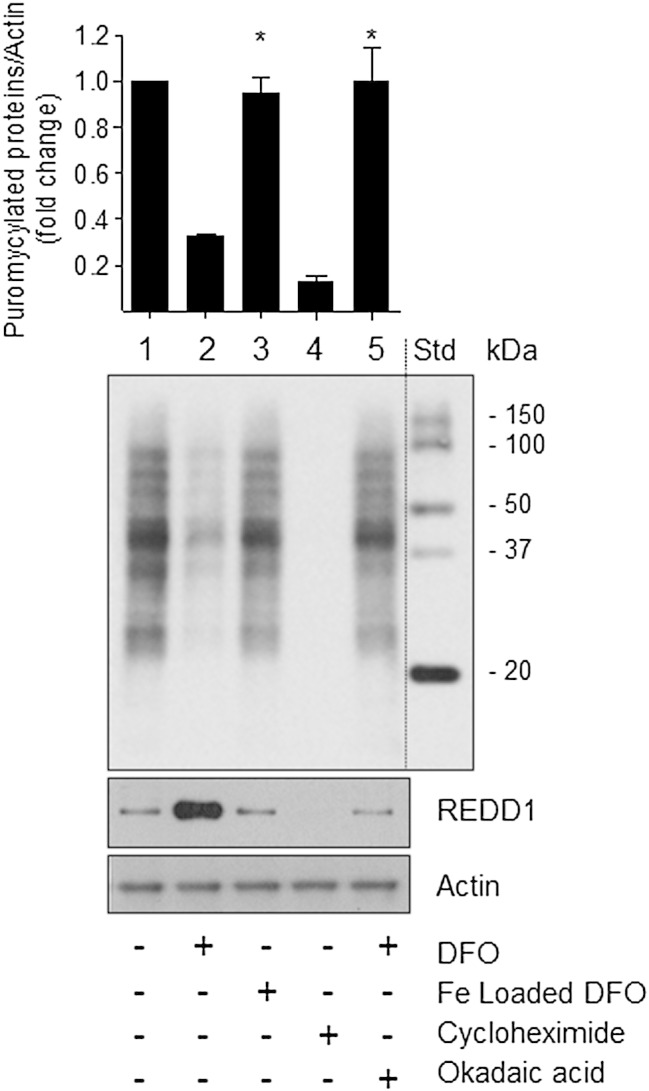
Effects of DFO-induced iron depletion upon protein synthesis in Caco-2 cells. Caco-2 cells were incubated with or without 100 μM DFO or 100 μM iron-loaded DFO for 16 h. In some experiments, DFO-treated cells were also incubated with okadaic acid (OKA, 100 nmol/l) during the penultimate 2 h iron depletion period with DFO, whereas a sub-set of non DFO-treated cells were incubated with 50 μg/ml of cycloheximide for 2 h. At the end of these respective treatments with DFO, iron-loaded DFO, okadaic acid or cycloheximide cells were incubated with 1 μM puromycin for 30 min before being lysed. Cell lysates were immunoblotted with antibodies to puromycin, REDD1 and actin. Quantification of puromycylated proteins from each treatment is presented as mean ± SEM from three independent experiments. Asterisks denote a significant change (*p < 0.05) from the value for DFO-treated cells (lane 2).

Impaired activation of the mTORC1/S6K1signalling pathway in iron depleted cells would be expected to impact negatively on protein synthesis. For analysis of protein synthesis we utilised an anti-puromycin antibody to assess incorporation of puromycin, a tyrosine-tRNA mimetic, into newly synthesised proteins. Fig. 9 shows that we were able to detect puromycylated proteins in cells held in normal media (Lane 1), but their abundance was significantly reduced in iron-depleted cells (Lane 2). This decrease was not apparent in Caco-2 cells that had been treated with iron loaded DFO (Lane 3) or those incubated with DFO, but in the presence of okadaic acid (Lane 5) — circumstances during which REDD1 expression was notably repressed (Fig. 9 lower panel). As an internal control, we also observed no labelling of proteins when Caco-2 cells were incubated with cycloheximide, which effectively precludes the incorporation of puromycin into nascent proteins by inhibiting translocation of mRNA on 80S ribosomes (Fig. 9, Lane 4). These changes in protein synthesis are consistent with effects that DFO has upon cellular growth and proliferation (Fig. 1).

## 4 Discussion

**Fig. 10:**
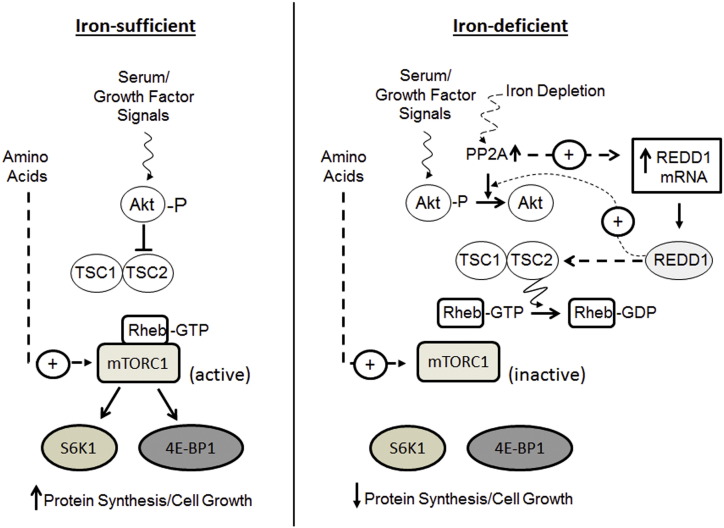
Scheme illustrating the mechanism by which mTORC1 signalling is regulated by iron depletion in Caco-2 cells.

The findings presented herein demonstrate that DFO-induced iron depletion promotes a loss in mTORC1-directed signalling in human intestinal Caco-2 cells and that this is associated with a marked reduction in protein synthetic capacity within these cells. Our data indicate that these changes are most likely driven by an increase in the expression of REDD1, a protein known to negatively regulate mTORC1 signalling [17], [38] and whose sustained upregulation in DFO-treated cells requires protein phosphatase 2A (PP2A) activity. Importantly, we demonstrate that PP2A inhibition or strategies that repress the increase in REDD1 expression alleviate the inhibition in mTORC1 signalling that is otherwise seen in iron depleted cells (Fig. 10 summarises our current working model of how iron deficiency impacts upon mTORC1 signalling).

Previous work using myeloid leukaemia cells identified REDD1 as one of a number of genes upregulated in response to cell treatment with the iron chelator, deferasirox [39]. The precise mechanism driving REDD1 expression under these circumstances has remained unclear, although given that iron chelators (such as DFO and deferasirox) can act as hypoxia mimetics, it is plausible that increases in REDD1 gene expression form part of a cellular hypoxic stress response. A central and well established feature of this response is the increased cellular abundance of the hypoxia-inducible transcription factor (HIF) which orchestrates expression of numerous target genes, including REDD1 [40], [41]. Consistent with this possibility, our analysis reveals that DFO treatment of Caco-2 cells is associated with increases in HIF-1α content. Nevertheless, we find that rates of cellular oxygen consumption and extracellular acidification (which reflect mitochondrial respiration and glycolytic activity, respectively) are not significantly altered in Caco-2 cells treated with DFO for 16 h (data not shown), which would suggest that cells retain normoxic status over the DFO treatment period used in our studies and that the increase in HIF-1α is not a consequence of hypoxia *per se*. A more likely explanation is that it arises through DFO-mediated inhibition of prolyl hydroxylases due to chelation of their Fe^2 +^ cofactor [34]. These enzymes help direct HIF-1α for proteosomal degradation and inhibition of their enzymatic activity will facilitate stabilisation/accumulation of HIF-1α and transcription of its target genes. In any case, the requirement for HIF-1α in mediating REDD1 gene expression in our iron depleted cells remains equivocal as we can “uncouple” effects of DFO on these two proteins with okadaic acid, which counters accumulation of REDD1 but not HIF-1α. This finding would seemingly negate a role for HIF-1 in the regulation of REDD1 expression in our DFO-treated cells and would be consistent with previous work suggesting that HIF-1 is neither necessary nor sufficient for hypoxia-induced downregulation of the mTOR pathway [42]. Nevertheless, since okadaic acid restrains the DFO-induced increase in REDD1 mRNA we cannot entirely exclude the possibility that our findings may at least partly reflect a requirement for PP2A to support DNA binding and/or transcriptional activation of the REDD1 gene in DFO-treated cells by HIF-1 or other known REDD1 transcription factors (*e.g.* ATF4, p53 and p63) [43], [44]. Such a possibility is not unprecedented in light of recent work showing that PP2A regulates binding of the sterol response element-binding protein (SREBP-2) to the sterol response element located within the promoter of the LDL receptor gene [45].

The notion that PP2A is an important regulator of the REDD1/mTORC1 signalling axis in iron depleted cells is supported by analysis of its phosphorylation status. Whilst phosphorylation of the catalytic subunit of PP2A on Tyr^307^ results in the inactivation of the enzyme [46], our studies reveal, for the first time, that DFO treatment of Caco-2 cells induces a significant reduction in phosphorylation of this site on PP2Ac. Phosphorylation of Tyr^307^ is targeted by *Src* kinase, whose activity was recently shown to be inhibited in iron-depleted cells [24]. Consequently, reduced Tyr^307^ phosphorylation of PP2Ac would be expected to enhance PP2A activity, which we suggest will have two important consequences in the context of the studies reported herein. First, elevated PP2A activity would support the DFO-induced increase in REDD1 gene expression and second, it will promote dephosphorylation/inactivation of Akt, which, in turn, will serve to inhibit mTORC1 signalling by maintaining TSC2-mediated repression of Rheb. From a mechanistic stand point, dephosphorylation of Akt depends upon the targeting of PP2A to Akt by REDD1. This latter proposition is based on the finding that in cells expressing REDD1 mutants that do not inhibit mTORC1 signalling the ability to coprecipitate PP2A with Akt and the loss in AktThr^308^ phosphorylation that normally results from this association is markedly reduced [18]. Whether such a mechanism operates independently or in conjunction with one in which REDD1 also promotes dissociation of TSC2 from inhibitory 14-3-3 proteins [17] to suppress mTORC1 signalling in our iron-depleted cells is currently unknown. It should be stressed that there is no direct evidence in support of direct REDD1/14-3-3 interactions and, indeed, structure–function studies do not favour them [47]. However, our findings clearly suggest that the cellular expression/abundance of REDD1 is a key determinant of mTORC1 signalling capacity in iron-depleted cells. This assertion is based upon the following lines of evidence. First, the reduction in mTOR signalling in iron depleted cells can be reversed upon resupplementing DFO-treated cells with iron, which not only attenuates REDD1 gene transcription but promotes rapid loss of REDD1 protein. Second, DFO-induced inhibition of mTORC1 is antagonised in cells in which REDD1 gene expression is halted either by use of a transcriptional inhibitor or REDD1 siRNA. Finally, since REDD1 is rapidly degraded by the proteasome, inhibiting proteosomal activity with MG132 results in its cellular accumulation and a concomitant reduction in mTORC1 signalling. Moreover, it is noteworthy that inhibition of mTOR signalling in DFO- or MG132-treated cells occurs despite the presence of amino acids and serum growth factors, thereby signifying that accumulation of REDD1 serves as a potent countermand that is capable of over-riding the stimulatory input normally conveyed by nutrients and growth factors upon mTORC1 [6].

Through its ability to phosphorylate its downstream effector molecules, S6K1 and 4E-BP1, mTOR exerts a positive influence upon mRNA translation. In line with this view, the loss in mTOR signalling that we observe in iron-depleted cells is associated with a marked inhibition in protein synthesis. This reduced protein synthetic drive is, we suggest, linked to induction of REDD1 as it can be mitigated by okadaic acid which suppresses REDD1 gene expression and thereby helps to maintain mTORC1 activity. Chronic exposure of Caco-2 cells to DFO also has a profound inhibitory effect on their growth and proliferative capacity. However, whilst reduced cell proliferation would be in accord with reduced protein synthesis and data indicating that mTOR activity is required for supporting cell cycle transit through G_2_/M [48], it is important to stress that growth inhibition of iron depleted cells is likely to only be partly explained by inhibition of mTOR signalling. Reduced iron availability will also inhibit the activity of ribonucleotide reductase [49], [50], an iron containing enzyme that crucially catalyses the formation of deoxyribonucleotides from ribonucleotides that are needed for DNA synthesis/replication and hence cell division. Furthermore, previous global transcriptomic studies utilising microarray technology and proteomic analyses of Caco-2 cells treated with DFO have reported significant changes in the expression of genes/proteins that fulfil essential roles in metabolism, cell cycle activity and apoptosis that occur in a manner that would serve to restrain growth and proliferation of iron-depleted cells [51], [52].

## 5 Conclusions

The present study shows that DFO-induced iron depletion prompts a significant increase in the expression of REDD1 that is associated with reduced mTOR signalling to key end-points such as protein synthesis, which, in turn, will impact negatively on cell growth and proliferation. The findings have important implications from the perspective of managing disorders linked to iron availability. They indicate that impaired mTOR signalling may contribute to atrophy and resultant dysfunction of the intestinal epithelium in individuals who are chronically iron deficient and therefore underscore the potential health benefits of dietary iron supplementation. The findings also signal the need to carefully monitor the use and efficacy of iron chelators. These compounds have been used clinically to treat iron-overload conditions (*e.g.* haemochromatosis, thalassemia [53]) and, by limiting iron availability for DNA synthesis, may also be effective anti-neoplastic agents that suppress growth and hyperproliferation of cancer cells [54]. However, our data would indicate that, unless iron chelators are used at concentrations that maintain iron stores within tight physiological limits or offer selective toxicity towards cancer cells, they may also inadvertently compromise function of normal cells through their effect not just on the mTOR pathway, but upon expression/activity of other proteins with key roles in signalling, metabolism and cell cycle biology.

## Conflict of interest

The authors declare no conflict of interest.
